# Treatment of Ankyloglossia: A Review

**DOI:** 10.3390/children10111808

**Published:** 2023-11-14

**Authors:** Alessandro Frezza, Fatima Ezeddine, Andrea Zuccon, Antonio Gracco, Giovanni Bruno, Alberto De Stefani

**Affiliations:** 1 Department of Neuroscience, School of Dentistry, University of Padova, 35122 Padova, Italy; alessandrofrezza94@gmail.com (A.F.); f.ezedine97@gmail.com (F.E.); andrea.zuccon@unipd.it (A.Z.); antonio.gracco@unipd.it (A.G.); giovanni.bruno.1@unipd.it (G.B.); 2 Department of Industrial Engineering, University of Roma Tor Vergata, 00133 Roma, Italy; 3 Department of Pharmacological Sciences, University of Padova, 35122 Padova, Italy

**Keywords:** ankyloglossia, lingual frenulum, laser, frenulotomy, frenulectomy

## Abstract

Aim: The aim of this narrative review is to analyze and compare the current scientific evidence regarding the diagnosis and treatment of hypertrophic lingual frenulum in preschool and school-age children. The treatments considered in this review are traditional surgical therapy, laser-assisted surgical therapy, and functional rehabilitation therapy. Materials and methods: A comprehensive literature review was conducted using the PubMed and PubMed Central search engines, considering articles published in the English language between 1 January 2000 and 30 June 2022. The bibliographic search was performed using the following keywords as search strings: “lingual”, “frenulectomy”, “frenulotomy”, “frenulum”, “ankyloglossia”, and “laser.” Results: A total of 14 articles were included in this review, including four prospective observational studies, one case–control study, three cross-sectional studies, four retrospective studies, and one randomized controlled trial. The data extracted from each article are summarized in a table. Conclusions: In the literature, there are still limited studies regarding the treatment of hypertrophic frenulum. No common indications for the treatment of ankyloglossia and universally used classification for lingual frenulum were found. Currently, clinicians prefer the use of a diode laser for treatment. This method offers several advantages over the use of a scalpel blade. Many studies agree on the usefulness of providing patients with myofunctional rehabilitation to improve lingual mobility, both prior to surgical therapy and in the postoperative period.

## 1. Introduction

Ankyloglossia (from the Greek words “ankylos”, meaning tied, and “glossa”, meaning tongue) is a congenital anomaly of the tongue characterized by a short lingual frenulum. This condition results in a limitation of tongue movements (partial ankyloglossia) or a fusion of the tongue to the floor of the mouth (total ankyloglossia) [1].

Ankyloglossia is more common in males compared to females, with a male-to-female ratio of 3:1. Its prevalence in the general population ranges from 4% to 10.7% [2,3], although these percentages are derived from studies conducted with different diagnostic criteria.

In neonates, ankyloglossia manifests with a prevalence of 5%, often as an isolated event. However, it can also be associated with malformation syndromes, such as Simpson–Golabi–Behmel syndrome, Opitz syndrome, Beckwith–Wiedemann syndrome, orofacial-digital syndrome, and cleft lip and palate [4].

Among the current diagnostic classifications for ankyloglossia, based on various anatomical and functional criteria, none has been universally accepted yet [2].

The ability of tongue elevation and protrusion is the most important quality for functions such as breastfeeding, feeding, speech, and development of dental arches [5,6].

Indeed, ankyloglossia is often associated with difficulties for infants in breastfeeding and bottle-feeding, limited tongue mobility, speech difficulties, malocclusion, and gingival recession. These challenges arise due to the restricted movement of the tongue caused by the short lingual frenulum [7,8].

Additionally, a short frenulum can greatly reduce tongue movements and create problems during swallowing. The limited mobility of the tongue can interfere with the proper movement of food or liquid during the swallowing process, leading to difficulties in ingesting and digesting food correctly. This can cause discomfort and compromise the efficiency of the feeding process [2,9]. During breastfeeding, a pathological frenulum can result in ineffective attachment of the baby, causing inadequate milk suction and persistent pain in the mother’s nipple. These are all factors that can negatively impact nutrition and lead to early weaning [7].

If the frenulum anomaly is severe enough to cause mechanical and functional limitations, surgical reduction of the frenulum (frenulectomy) is indicated, followed by speech therapy for immediate rehabilitation of the tongue muscle.

Ankyloglossia often leads to difficulties in pronouncing certain consonants and sounds, such as /z/, /s/, /t/, /d/, /l/, /sh/, /ch/, /th/, /dg/, and especially the letter /r/ [8,10].

Speech therapy, in conjunction with frenulectomy, frenulotomy, or frenuloplasty, can be a therapeutic option to improve tongue mobility and consonant pronunciation. By addressing any limitations in tongue movement and working on specific speech exercises, speech therapy can help individuals with ankyloglossia improve their articulation and overall speech abilities [11].

However, there is not always a direct relationship between hypertrophic lingual frenulum and language limitations. Many children and individuals with ankyloglossia are able to compensate for this reduced tongue mobility and do not appear to suffer from any speech-related issues. The evidence demonstrating that ankyloglossia and abnormal tongue position can influence skeletal development and be associated with malocclusions is limited [12].

Some studies, however, suggest that a high-arched palate and an elongated soft palate are associated with hypertrophic lingual frenulum [6].

Localized gingival recession on the lingual aspect of mandibular incisors, in some cases, is precisely due to an anomalous attachment of the lingual frenulum that causes the recession. As with most periodontal conditions, eliminating plaque-induced gingival inflammation can minimize gingival recession without any surgical intervention. However, when recession persists even after oral hygiene management, surgical intervention may be indicated [2].

Guilleminault [13] states that a short lingual frenulum is a frequent phenotype for pediatric sleep apnea. Many studies in the literature have shown an improvement in sleep quality in patients who underwent frenulectomy, both with laser and scalpel procedures [4,14,15,16].

The objective of this narrative review is to analyze and compare the current scientific evidence regarding the diagnosis and treatment of hypertrophic lingual frenulum in preschool and school-age children. The treatments considered in this review are traditional surgical therapy, laser-assisted surgical therapy, and functional rehabilitation therapy.

## 2. Materials and Methods

An extensive literature review was conducted using the search engines PubMed and PubMed Central, considering articles published in the English language between 1 January 2000 and 30 June 2022. The bibliographic search was performed using the following keywords: “lingual”, “frenulectomy”, “frenulotomy”, “ankyloglossia”, and “laser.” The string used was “lingual and (frenulectomy or frenulotomy of ankyloglossia).” The results were filtered by the use of the word “laser.”

The research focused on the PICO question illustrated in Table 1. Clinical studies that addressed surgical or laser-assisted interventions were analyzed, while literature reviews, letter reports, non-in vivo studies, and surveys were excluded from the literature search.

The identification, screening, and inclusion phases were conducted by two independent researchers. In case of disagreement between the two, only studies with unanimous consensus were included.

**Figure 1:**
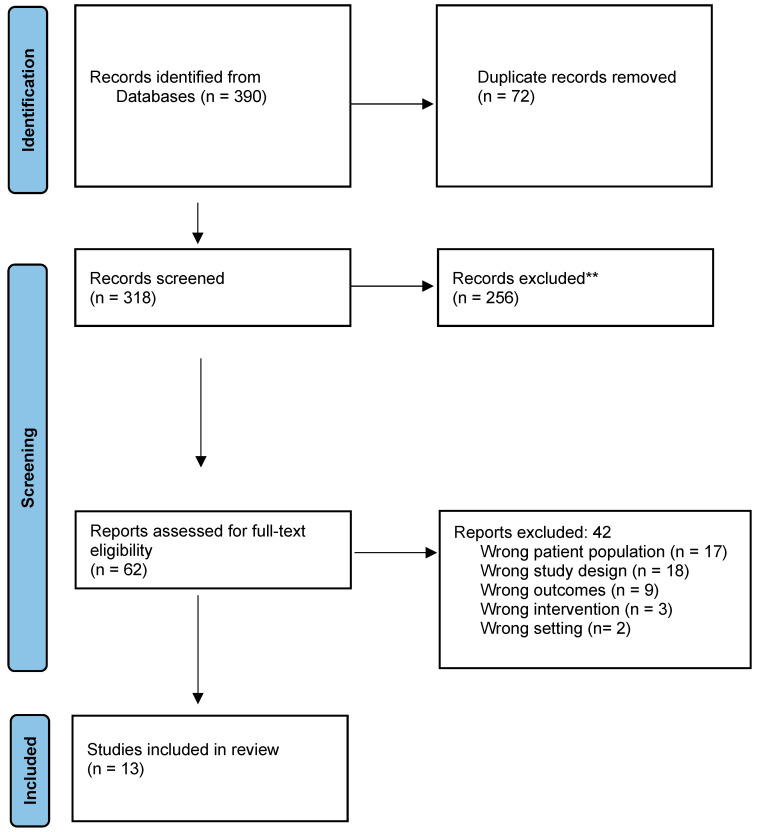
PRISMA flow chart. ** records exluced according with the inclusion and the exclusion criteria.

In the initial identification phase, articles that could potentially be included in the review were selected, and any duplicates were removed. This was followed by a screening phase, in which articles that did not appear to align with the purpose of the research based on the title and abstract were excluded. Subsequently, after reading the full text of the remaining articles, a total of 13 relevant studies were included in our investigation (Figure 1).

The quality of the studies was evaluated using methodological quality criteria (Table 2) adapted from the CONSORT statement and Jadad quality assessment [17]. The selected studies were independently scored by the two reviewers. In case of disagreement, the scoring was assigned through discussion. Each article received a score out of 11 points based on the methodological quality criteria and classified as good if higher than 9 points, moderate if between 7 and 9 points, and poor if lower than 7 points. The results of the studies were entered into Excel tables for comparison. The obtained data were categorized by author, year of publication, study type, population sample characteristics (size, gender, and age), diagnostic method, surgical therapy, rehabilitation therapy, and obtained results.

## 3. Results

A total of 13 articles were included in this review, including four prospective observational studies, one case–control study, three cross-sectional studies, four retrospective studies, and one randomized controlled trial.

Table S1 presents the selected articles and their results (see Supplementary Materials).

## 4. Discussion

Epidemiology: The selected prevalence studies in this review report contrasting results. Jamilian et al. [20] reported a higher incidence of ankyloglossia in males, in line with a study conducted in India by Pavithra et al. [21] on a population of 700 children, although the gender difference was not statistically significant. On the contrary, Ruffoli et al. [18] did not find differences between males and females. In general, the literature reports a higher prevalence of ankyloglossia in males, as also demonstrated by studies conducted by Jorgenson et al. [27], Messner et al. [28], and Ballard et al. [29].

Prevalence: A prevalence of 16.4% was reported in the population in India by Pavithra et al. [21], with the majority being classified as grade I (48%), followed by grade II (30%), grade III (15%), and grade IV (8.85%), according to the Kotlow classification. This incidence was found to be higher compared to other studies, including those conducted by Messner et al. (4.8%) [28], Hogan et al. (10.7%) [30], Ballard et al. (3.2%) [29], and Friend et al. (12.8%) [31].

Differences could be attributed to different classification methodologies and measurements.

Classification: Currently, there is no unanimous consensus in the literature regarding the classification of ankyloglossia, which can also explain the differences between studies and the difficulty in comparison. Numerous methods have been proposed, such as those by Kotlow et al. [32], Garcia Pola et al. [33], Horton et al. [34], and Ruffoli et al. [18].

One of the most commonly used systems for the classification of ankyloglossia is the Kotlow classification. Kotlow was the first to propose an anatomical criterion for classification, determining that a free tongue has a frenulum within the normal range if it measures equal to or greater than 1.6 cm, a value also confirmed by the Ruffoli classification. The same study by Jamilian [20] used the criteria of Ruffoli, observing a sample of 300 children, and found that only children with a lingual frenulum <1.5 cm had limited tongue mobility. However, only children with a frenulum length <0.7 cm had abnormal language patterns and inadequate tongue movement.

The study by Pavithra et al. [21] is an interesting analysis of a group of individuals aged 9 to 17 years, in which the lingual frenulum has completed its development and is investigated in relation to orthodontic concerns, such as crowding and lower anterior relapse.

Surgical and rehabilitative therapy: The study by Tancredi et al. [26] highlights the operative and postoperative advantages of treating hypertrophic lingual frenulum using diode laser therapy, evaluating the extent of postoperative healing and differences in pain perception compared with traditional surgical methods.

Firstly, patients treated with laser therapy reported significantly reduced pain compared to those treated with traditional methods, both immediately after surgery and one week postoperation. Additionally, the laser resulted in better tissue healing. These advantages are complemented by other benefits, including minimal bleeding, a “clean” surgical field, no need for sutures, and no requirement for anti-inflammatory or antibiotic therapy. Laser treatment is also faster.

Unlike a scalpel blade, the laser does not cut through the structures that make up the lingual frenulum (collagen and elastic fibers) but rather causes denaturation and coagulation. Therefore, it can be concluded that laser-assisted intervention offers numerous advantages over conventional surgical techniques. This is also confirmed by other recent studies. In particular, Nammour et al. [35] focused on the significant advantage of not having to suture traumatized tissues during the procedure. However, Brignardello-Petersen [36] and Viet et al. [37] highlighted the reduction in patient discomfort, shorter operation time, and reduced amount of anesthesia required as benefits of using a diode laser compared to traditional surgery.

However, Tancredi’s study has several limitations. Firstly, it was not possible to divide the participants into the two groups in a proportionate manner, controlling variables such as ethnicity, oral hygiene level, socio-economic conditions, and other determining factors equally. Secondly, the retrospective nature of the study limited the collection of relevant data regarding postoperative improvement, such as tongue mobility, speaking ability, chewing, and swallowing. Additionally, the sample size was small, and there was an error in the pain measurement index due to the use of a numeric rating scale (NRS), which tends to report extreme values rather than a visual-analog-scale (VAS) index. The study by Komori et al. [23] also highlights the effectiveness of using a laser in the treatment of lingual frenulum. In this case, the laser used is the CO_2_ laser, which has been shown to be useful in treating frenula. It is simple to use and safe, providing good postoperative results. The use of the CO_2_ laser ensures reliable hemostasis and early healing, as the surface and depth of resection are reduced compared with conventional laser use. In the study by Haytac et al [38], they demonstrate how patients treated with the use of this laser had significantly lower pain values on the visual analog scale (VAS) compared with those treated with traditional methods. However, a significant issue in these studies is the limited sample sizes.

Fioravanti et al. [16] evaluated the effectiveness of lingual frenulectomy using a diode laser to improve both the length of the frenulum and the obstructive-sleep-apnea-syndrome (OSAS) index in pediatric patients. In patients treated with the laser, there was a significant improvement in tongue mobility and the OSAS index. This study has demonstrated that lingual frenulectomy with laser is a valid method for treating OSAS in pediatric patients.

In addition to this aspect, the laser offers numerous benefits, including adequate hemostasis, reduced operating time, easier access, surgical site disinfection, precise incision, minimal tissue damage, more effective tissue healing, reduced inflammation, better control of postoperative pain, and greater acceptance and compliance, especially in pediatric patients. Reddy et al. [39] compared three techniques for the treatment of lingual frenulum: electrosurgery, cold blade, and diode laser. After follow-up periods of 7 days and 30 days, better tissue healing was observed in patients treated with the laser, while the other techniques reported higher indices of pain and swelling. Also, Derikvand et al. [40] have demonstrated positive factors of using laser technology in terms of healing and postoperative complications. Furthermore, Barot et al. [41] reported an improvement in tongue mobility and speech in patients treated with a laser. The absence of traditional surgical instruments and the hemostatic effect of the laser allow for bloodless surgeries without the need for sutures, which would be uncomfortable during the postoperative period, especially when performing myofunctional exercises.

These elements are particularly useful in young patients who may experience more anxiety towards surgery compared with adults. Children are more accepting of the laser; the sight of the beam of light during the preparatory phase of the procedure generates curiosity and can help increase patient compliance during the intraoperative phase.

Baxter et al. [15] have shown that frenulectomy using a CO_2_ laser leads to improvements in lingual functions (speech, eating, and sleeping), with more significant results when there is a complete release of the frenulum. The study emphasizes the importance of early detection and assessment of functional limitations of the lingual frenulum, including difficulties in speech, chewing, and sleep issues, in order to develop individualized care plans for myofunctional therapies.

Following surgery and myofunctional exercises, communication became easier, and parents and other children found it easier to understand words. There was also an increase in speech rate, improved production of previously difficult sounds, and the production of new words by children with language delays. Parents also noticed positive changes in their children’s feeding habits, as they ate more quickly, were less demanding, spit out food less frequently, and chewed more effectively. There were also improvements in sleep quality, as children were less likely to sleep in odd positions, kick, or move during the night. They slept more deeply, woke up less fatigued, and had reduced mouth breathing, teeth grinding, and snoring. Parents also noted a reduction in neck pain, headaches, open-mouth breathing, and vomiting reflex, as well as decreased hyperactivity, inattention, reflux, and constipation.

Regarding traditional surgical therapy, there are two methods: simple frenulotomy and Z-plasty frenuloplasty.

Kim et al. [25] compared these two traditional surgical techniques: Z-plasty frenuloplasty and simple frenulotomy. The Z-plasty frenuloplasty was performed using a no. 15 blade, while the simple frenulotomy was performed using an electrosurgical device. There were no statistically significant differences between the two groups for preoperative values of ankyloglossia and speech tests.

Both surgical methods were found to be effective in correcting ankyloglossia and improving speech.

In the literature, there is no ideal surgical treatment procedure for ankyloglossia. Several studies have attempted to demonstrate the effectiveness of various surgical techniques by comparing them to each other. Ito et al. [42] studied the procedures of frenuloplasty and frenulotomy to correct articulatory disorders following surgery. Yousefi et al. [43] reported that Z-plasty shows superiority in improving articulation compared to simple release. Z-plasty is a surgical procedure that releases contractile or scar tissue based on the suturing technique used in plastic surgery. However, this surgical procedure is usually challenging to perform under local anesthesia in children and is therefore performed under general anesthesia or conscious sedation. It is important to demonstrate that the surgical outcomes of Z-plasty with four flaps are superior to simple frenulotomy, which can be performed under simple local or regional anesthesia. In the literature, postoperative complications are not commonly reported, but events such as excessive bleeding, upper airway collapse, infection, diathermy burn of the lip, ulcer under the tongue, lingual dysfunction, swallowing abnormalities, and reattachment of the surgical site have been reported. In the study by Kim et al. [25], no specific complications were observed [25].

Zaghi et al. [14] treated patients with pathological lingual frenulum through surgical frenulotomy with a scalpel combined with myofunctional rehabilitation. From their results, it was found that almost all patients (91.1%) were satisfied with the treatment they received and reported benefits in tongue mobility, nocturnal grinding, breathing, and sleep. In total, 45% of patients experienced postoperative pain. Linguoplasty in conjunction with myofunctional therapy can be an effective treatment for mouth breathing, snoring, grinding, and myofascial tension. However, in this case as well, the traditional treatment caused a significant percentage of postoperative pain.

An increasing number of healthcare practitioners are seeking evidence-based information in the literature for the treatment of ankyloglossia. However, few researchers are publishing articles on this topic. Most of the published articles consist of limited clinical cases and case series. There are available larger cohort studies on frenulectomy techniques for infants related to breastfeeding [44]. However, research on the treatment of ankyloglossia among children [22], adolescents [45], and adults is still limited.

In the article by Ferras Amat et al. [22], the importance of myofunctional rehabilitation after traditional surgical treatment of the frenulum is demonstrated. In fact, 96% of patients treated with frenulectomy and rhomboid flap with dissection of the genioglossus muscles achieved non-pathological degrees of lingual frenulum after orofacial rehabilitation.

In the literature, various traditional surgical techniques are described for releasing the tongue from the low attachment of the frenulum, but none of these techniques are widely accepted as a treatment guideline.

Heller et al. [46] recommend the “Z-plasty” technique. Their data indicates that the four-flap Z-plasty is superior to the horizontal-to-vertical frenuloplasty in achieving tongue lengthening, protrusion, and improvement in speech articulation for patients with symptomatic ankyloglossia. Messner et al [10] found that the surgical intervention to release the frenulum is a safe and effective procedure that results in improved tongue mobility and often better speech articulation. Some articles in the literature suggest that some children with hypertrophic lingual frenulum are able to develop normal language skills by compensating for the reduced mobility of the tongue tip. However, in certain cases, the pathological lingual frenulum can be symptomatic, causing articulation errors or difficulties with speed and range of speech. The pronunciation of consonants T, D, Z, S, N, and L can be influenced by reduced mobility of the tip of the tongue, but the percentage of patients who experience a language disorder based on reduced tongue mobility remains uncertain, and there is no method to predict in early age which patients will require treatment. The study by Messner et al. demonstrates that a certain number of children with ankyloglossia can have normal language despite reduced tongue mobility. However, a significant percentage of children with ankyloglossia (71%) will have articulation difficulties related to the tongue issue.

A study contradicting the usefulness of surgical therapy for tongue-tie is that of Daggumati et al. [24]. In this study, they compared 220 patients divided into two groups: the first group received surgical treatment for tongue-tie, while the second group only underwent functional rehabilitation exercises. From the results, it was found that there was no statistically significant difference in language quality between children with surgically treated ankyloglossia and those who underwent conservative treatment. However, patients treated with frenulectomy showed less difficulty in performing assigned linguistic tasks. Therefore, according to these authors, a conservative approach to ankyloglossia may be feasible in the early stages.

Another article in agreement with this study is that of Dollberg et al. [9]. In this study, they demonstrated that there was no qualitative difference in word articulation in children who underwent surgical treatment for tongue-tie compared with those of the same age who did not have surgical intervention for ankyloglossia or who did not have a pathological frenulum. However, the limitation of this study is the small sample size, as they only studied 23 children. Other studies in the literature have shown conflicting results on the quality of language after surgical frenulectomy. Very often, frenulectomy is performed without a precise indication for treatment, as it is a simple and quick procedure, especially with the use of new laser-assisted technologies. Daggumati et al. state that the simplicity of this operation should not be a justification for treating any type of patient, but there should be an appropriate indication.

The study by Daggumati also has its limitations, and its results must be carefully considered, as the sample size is modest, although it is still the largest study in the literature for the group of patients who did not undergo surgical treatment. Furthermore, the perception of language quality is entirely subjective and heavily relies on the responses of parents to questionnaires.

One of the most significant limitations of this review that remains is the small number of publications related to tongue-tie. All the studies analyzed are in the English language and had a limited number of participants in the studied population samples, and many of those studies used different classifications for tongue mobility.

## 5. Conclusions

Currently, there are still few published articles regarding the treatment of pathological lingual frenulum. There are no guidelines available, and there is no universally accepted classification.

For the treatment of lingual frenulum, it can be concluded that clinicians prefer the use of a diode laser due to its numerous advantages over the use of a scalpel blade.

Many studies agree on the usefulness of incorporating myofunctional rehabilitation for patients to improve lingual mobility, both before and after surgical therapy. The development of adequate lingual mobility can contribute to improving the patient’s quality of life, especially if the problem is detected early, as it can prevent situations such as palatal contractions, dental crowding, and sleep-related breathing disorders.

It is desirable and significant to continue conducting further in-depth studies on this topic and advise healthcare professionals to pay attention to the morphological characteristics of the frenulum and limitations of the tongue during early visits in order to identify children affected by ankyloglossia as early as possible.

## Figures and Tables

**Table 1:** PICO questions.

P	Individuals Aged 1–18 years
I	Frenulectomy or frenulotomy of lingual frenulum
C	Absent
O	Results of the surgical approach and functional rehabilitation therapy
S	Cohort studies, retrospective studies

**Table 2:** Jadad quality assessment.

Authors	Items for Methodological Quality Criteria											Total Score	Methodological Quality of the Study
	A	B	C	D	E	F	G	H	I	J	K		
Messner et al. (2002) [10]	0	1	0	1	0	0	0	0	1	1	1	5	POOR (<7)
Ruffoli et al. (2005) [18]	0	1	0	1	0	0	0	1	1	1	1	6	POOR (<7)
Srinivasan et al. (2013) [19]	0	1	1	1	0	0	0	1	1	1	1	7	MODERATE (7 < x < 9)
Jamilian et al. (2014) [20]	0	1	0	1	0	0	0	0	1	1	1	5	POOR (<7)
Pavithra et al. (2014) [21]	0	1	1	1	0	0	0	0	1	1	1	6	POOR (<7)
Elvira Ferrés-Amat et al. (2016) [22]	0	1	0	1	0	0	0	0	1	1	1	5	POOR (<7)
Komori et al. (2017) [23]	0	1	1	1	1	0	0	0	1	1	1	7	MODERATE (7 < x < 9)
Daggumati et al. (2019) [24]	0	1	1	1	1	1	0	1	1	1	1	9	GOOD (>9)
Zaghi et al. (2019) [14]	0	1	1	1	1	1	0	1	1	1	1	9	GOOD (>9)
Baxter et al. (2020) [15]	0	1	1	1	1	1	0	1	1	1	1	9	GOOD (>9)
Kim et al. (2020) [25]	1	1	1	1	1	1	1	1	1	1	1	11	GOOD (>9)
Fioravanti et al. (2021) [16]	1	1	1	1	1	1	1	1	1	1	1	11	GOOD (>9)
Tancredi et al. (2022) [26]	0	1	1	1	1	1	0	1	1	1	1	9	GOOD (>9)
**Sr No.**	**Item**												**Score**
A	Design of randomised clinical trial												1
B	Eligibility criteria for study particpants												1
C	Sample size determination												1
D	Details about clinical diagnostic criteria												1
E	Ethical consideration												1
F	Method of blinding												1
G	Methods and type of randomization												1
H	Description of recruitment period and follow-up												1
I	Withdrawals and dropouts												1
J	Clearly defined outcomes												1
K	Appropriate statistical analysis												1
	Total score												11

## Data Availability

Not applicable.

## Supplementary Materials

The following supporting information can be downloaded at https://www.mdpi.com/article/10.3390/children10111808/s1, Table S1: Studies included in review.
