# Supplementary material for: Treatment of Ankyloglossia: A Review
Source: Children (Basel). 2023 Nov 14;10(11):1808. doi: 10.3390/children10111808 (PMC10670877; doi:10.3390/children10111808)
Supplement: Supplementary file 1 [file children-10-01808-s001.zip › children-2644559-supplementary.pdf]

Table S1: Studies included in review.

Author	Study	Sample Size	Age	Diagnosis	Surgical Therapy	Rehabilitation Therapy	Results
**Messner et al. (2002) [10]**	Prospective observational study	*n* = 30 (19 M + 11 F)	1-12	Measurement of lingual mobility in elevation and protrusion. The interincisal distance during the maximum mouth opening when the tip of the tongue touches the retroincisive papilla and the maximum protrusion as the distance of the tongue from the lower teeth were measured. Questionnaire to be completed to check the quality of pronunciation	Frenuloplasty and traditional frenulotomy with the use of scalpel and stitches	-	Surgery to release the lingual frenulum is a safe and effective procedure that involves improved lingual mobility and often better articulation of words.
**Ruffoli et al. (2005) [18]**	Prospective observational study	*n* = 200 (100 M + 100 F)	6-12	Ruffoli classification by degree of ankyloglossia and lingual mobility<br>Pronunciation of 21 phonemes for speech therapy	-	-	No gender differences in prevalence
**Srinivasan et al. (2013) [19]**	Case-Control study	*n* = 117 (N/A)<br>Test *n* = 57<br>Controllo *n* = 60	N/A (mean age 13y)	Kotlow classification<br>SNA, SNB, ANB, Go-Gn-SN angle, FMA, maxillary and mandibular intercanine and intermolar amplitude, tooth-arch discrepancy, overbite	-	-	Most patients had moderate ankyloglossia and ankyloglossia was more common in males.<br>The most frequently seen skeletal class in patients with ankyloglossia was class I, followed by class II and class III.<br>Patients with ankyloglossia had arches with reduced intercanine and intermolar diameters compared to the control group.<br>The downward rotation of the mandibular plane and the open bite are as severe as the severity of the ankyloglossia.
**Jamilian et al. (2014) [20]**	Cross-Sectional Study	*n* = 300 (150 M + 150 F)	7-14	Kotlow criteria for lingual mobility<br><br>Ruffoli classification by degree of ankyloglossia	-	-	Higher prevalence in men<br>Subjects with lingual frenulum < 1.5 cm had limited lingual movement
**Pavithra et al. (2014) [21]**	Cross-Sectional Study	*n* = 700 (N/A)	9-17	Kotlow criteria for degree of ankyloglossia	-	-	16.4% degree of ankyloglossia, especially Kotlow's grade I and III<br>No statistically significant differences between general population and special people<br>Prevalence in the male gender (not statistically significant)
**Elvira Ferrés-Amat et al. (2016) [22]**	Cross-Sectional Study	*n* = 101 (63 M + 38 F)	4-14	Classification on lingual mobility proposed by the authors on 5 degrees of severity. Grades 4 and 5 represent reduced mobility and indication for treatment.	Frenulectomy with traditional method with the use of the scalpel and with rhomboid plastic and dissection of the	Orofacial rehabilitation started 1 week before the operation and then continued	28% of treated patients reached grade I and II of lingual mobility even after the first phase of rehabilitation. 96% of patients achieved full lingual mobility after surgical therapy and myofunctional therapy. 6% of patients experienced post-operative complications(4 cases of traumatic bite, 1 of post-

					genioglossus muscle + suture		operative haemorrhage and 2 cases of infections). Only in 4 cases the lingual mobility did not improve even after surgical therapy.
**Komori et al. (2017) [23]**	Retrospetctive Study	*n* = 35 (20 M + 15 F)	0-14	Ito classification to evaluate lingual frenulum and Rui classification to evaluate upper labial frenulum.	Lingual frenulectomy (27 patients) and labial frenulectomy (8 patients) with the use of CO2 laser from 2 to 5 W for up to 60 sec.	-	There were no intraoperative problems and the surgery was fast and safe. No patients complained of post-operative problems such as pain or bleeding. Readhesion of the frenulum occurred only in one subject. In this observational study, the average age of lingual frenulum treatment was 6 years. This study demonstrated the usefulness of the CO2 laser in the treatment of frenuli. Its use is simple and a safe method to obtain good results in the post-operative period.
**Daggumati et al. (2019) [24]**	Retrospetctive Study	*n* = 77 (52 M + 25 F)<br>- Test *n* = 46 (20 M + 11 F)<br>-Controllo *n* = 31 (14 M + 32 F)	0-18	Ankyloglossia: protrusion of the tongue beyond vermilion edge<br>No elevation of the apex of the tongue<br><br>Survey 15 questions to caregivers.	Test: frenucletomy (N/A type of intervention)	65% case<br>71% control	There are no statistically significant differences in language difficulty and language mobility between the test group (patients who performed frenulectomy) and control.<br>Surgical patients reported fewer difficulties in lingual function than control.
**Zaghi et al. (2019) [14]**	Retrospetctive Study	*n = 348* (N/A)	3 - 79	-	Frenuloplasty with the use of scalpel + suture.	All patients treated with surgery underwent myofunctional therapy. The exercises were started 1 month before the intervention and then continued up to 1 year after the intervention	91.1% of patients were satisfied with the therapeutic treatment received. Patients reported the following benefits: improvement of lingual mobility, reduction of nocturnal grinding, improvement of nasal breathing and sleep quality. 45% of patients reported episodes of post-operative pain. The authors conclude that lingual frenuloplasty is an effective treatment for oral breathing, snoring, nocturnal grinding and reducing oro-myophacccial tension..
**Baxter et al. (2020) [15]**	Prospective observational study	*n* = 37 (23 M + 14 F)	1-13	Kotlow's classification was used during the first visit to subdivide patients' lingual frenules	Frenulectomy using CO2 laser	Recommended myofuntial exercises 2-3 times a day after surgery	This study showed that CO2 laser frenulectomy resulted in improvements in lingual functions (talking, eating and sleeping). The best results were noticeable when the total release of the frenulum was performed. Healthcare professionals should always check for functional limitations of the lingual frenulum in the presence of difficulties in speech, sleep and chewing. Very useful are the use of individual care plans for myofunctional therapies.

**Kim et al. (2020) [25]**	Prospective randomized study	*n* = 37 (23 M + 14 F)	3-7	Kotlow classification + U-TAP Korean phonation evaluation test	18 patients performed traditional frenulotomy using scalpel + stitches<br>19 patients performed Z-shaped frenuloplasty using scalpel + stitches	-	All patients, with the exception of one subject, achieved normal lingual mobility (Kotlow's Class I) in the post-operative period. In only one case did the lingual function not improve. No statically significant differences in the two comparison groups for the surgical techniques used. Both groups showed similar results with regard to improving lingual mobility and phonation.<br>In conclusion, both surgical methods were effective in correcting ankyloglossia and improving phonation..
**Fioravanti et al. (2021) [16]**	Randomized controlled trial	*n = 32* (18 M + 14 F)	4-13	Kotlow classification + Ruffoli classification + AHI	The study group was treated by frenulectomy with the use of diode laser	Myofunction exercises were performed by patients in the study and postoperative group and control group	The study group shows a greater increase in all the scores analyzed (lingual mobility + OSAS entities) after 1 month after surgery. The control group also had improvements in scores, although less significant.<br>Patients in the study group had a more significant improvement in OSAS index than control patients. This study demonstrated how laser-assisted lingual frenulectomy can significantly improve OSAS severity in pediatric patients.
**Tancredi et al. (2022) [26]**	Retrospetctive Study	*n* = 61 (29 M + 32 F)	8-12	Kotlow classification	Group A was treated by traditional frenulectomy using a scalpel. Group B was treated by laser-assisted frenulectomy with the aid of diode laser	Myofunctional exercises were prescribed to both groups in the postoperative	From the questionnaires completed by the patients of the two groups it was deduced that the post-operative pain reported by the patients of group B was much reduced compared to patients of group A and the laser determined a better healing of the tissues.<br>The authors recommend the use of laser compared to the traditional technique for the reasons given above and because it allows the absence of bleeding, a clean operating field, the non-need to suture and to prescribe anti-inflammatory and antibiotic therapy. The operating time with the laser is significantly decreased compared to that with the traditional technique.
